# Supplementary material for: Nationwide Survey on Seasonal Influenza Vaccination among Health Care Workers during the COVID-19 Pandemic in Greece: Determinants, Barriers and Peculiarities
Source: Int J Environ Res Public Health. 2023 Jun 28;20(13):6247. doi: 10.3390/ijerph20136247 (PMC10341827; doi:10.3390/ijerph20136247)

Supplementary materials

File S1. questionnaire

UNIVERSITY OF THESSALY
SCHOOL OF HEALTH SCIENCES
FACULTY OF MEDICINE
DEPARTMENT OF HYGIENE & EPIDEMIOLOGY

QUESTIONNAIRE ON KNOWLEDGE, ATTITUDES AND PRACTICES OF HEALTH CARE PROFESSIONALS RELATED TO SEASONAL INFLUENZA VACCINATION

GENERAL SECTION

1. Age: …….. years
2. Gender: □ Male □ Female
3. Marital status (indicate with 'X'): □Married □Unmarried □Other (please specify)……………

4. Education level (indicate with 'X'):
□ High school □ Institute of Vocational Training (IEK)
□ Technological Educational Institute (TEI) □ Higher Education Institute/University (AEI) □ Master /Doctoral

5. Health care profession (indicate with 'X'):
□ Doctor (please indicate specialization ……………………………) □ Nurse
□ Medical Laboratory worker □ Other (please specify).............................

6. Sector of employment (indicate with 'X'):
□ Private hospital □ Public hospital
□ Health centre (K.Y.) □ Community-based primary health unit (To. M.Y)

7. Health District (Υ.ΠΕ) of employment: ……………………………………………………………………………

8. Regional Unit of employment: …………………………………………..

9. Department of employment (indicate with 'X'): (please do not respond if you work at a health centre or community-based primary health unit)
□Clinical □Laboratory □ Other (please specify).............................

10. Section of employment (indicate with 'X'): (please do not respond if you work at a health centre or community-based primary health unit)
□ Pathology □ Surgery
□ Laboratory □ Other (please specify).............................

11. Years of practice: ....……………

SPECIFIC SECTION

12. Do you belong to a vulnerable/high risk group due to your medical history? (indicate with 'X'):
(cardiovascular disease, respiratory disease, diabetes, immunosuppression, cancer, pregnancy etc.)
□ YES □ NO

13. Do you live with older individuals or individuals belonging to a vulnerable/high risk group due to their medical history? (indicate with 'X'):
(cardiovascular disease, respiratory disease, diabetes, immunosuppression, cancer, pregnancy etc.)
□ YES □ NO

14. Please circle the choice that best indicates your response.

	Completely agree	Agree	Neither agree nor disagree	Disagree	Completely disagree

The HPV vaccine is recommended for all males up to 18 years of age in the country.	1	2	**3**	4	5
After the flu vaccination, certain foods are not permitted to be consumed for a period of 24 hours.	1	2	**3**	4	5
One of the contraindications of the flu vaccine is an allergy to eggs.	1	2	**3**	4	5

15. Please circle the choice that best indicates your response.

	**Completely agree**	**Agree**	**Neither agree nor disagree**	**Disagree**	**Completely disagree**
Vaccinations are an important tool for the protection of public health and in particular of health professionals and workers in the health sector	1	2	**3**	4	5
Natural immunity acquired via disease is always preferable to immunity acquired via vaccination.	1	2	**3**	4	5
Many vaccines often have serious side effects.	1	2	**3**	4	5

16. Are you the parent/guardian of one or more children? (indicate with 'X'):

□ YES □ NO

If **YES,** do you adhere to the child vaccination program suggested by the National Vaccination Program in the country? (please circle the answer of your choice)

□ YES, I vaccinate my children according to the National Vaccination Program

□ I select and carry out some vaccinations □ I do not vaccinate my children

17. Have you been vaccinated with the seasonal flu vaccine? (indicate with 'X'):

□ YES □ NO

If not, please indicate why (more than one response can be selected)

□I do not have time □ Apathetic

□I use homeopathic remedies □I do not think I am at risk

□Fear regarding vaccine safety □ Other (please specify)..............................

18. Which channels do you use to keep informed about the COVID-19 pandemic and the SARS-CoV-2 vaccine, and how often? (please circle the option that represents your answer)

	**Always**	**Often**	**Rarely**	**Never**
Television	1	2	3	4
Social media channels (Facebook, Twitter, Instagram etc.)	1	2	3	4
Newspaper (in print or electronic versions)	1	2	3	4
General interest publications/journals (in print or electronic versions)	1	2	3	4
Medical articles in journals (in print or electronic versions)	1	2	3	4
Committee for infectious diseases at health facility	1	2	3	4
General interest websites	1	2	3	4
Website of the Hellenic National Public Health Organization (NPHO)	1	2	3	4
Website of the Hellenic Ministry of Health	1	2	3	4
Please specify other ………………………………………………………………………….…….	1	2	3	4

19. Please circle the response below that represents your answer.

	Completely agree	Agree	Neither agree nor disagree	Disagree	Completely disagree
Some of the vaccines against SARS-CoV-2 which are approved and used in the country are based on mRNA technology	1	2	3	4	5
The dosage regimen of the vaccines against SARS-CoV-2 includes 3 doses	1	2	3	4	5
There is evidence that mRNA technology interferes with the DNA of cells	1	2	3	4	5

20. Have you been or will you be vaccinated with any of the vaccines against the SARS-CoV-2 virus which causes COVID-19, which have received the necessary approvals from the European Medicines Agency and the National Medicines Agency? (indicate with 'X'):

□ YES □ NO

If not, please indicate why (**indicate with 'X'**) (more than one response can be selected)

- □ I do not have time
- □ Apathetic
- □ Fear regarding vaccine safety
- □ I do not think I am at risk
- □ I need further information in order to make a decision

Other (please specify) …………………………………..…………………………………….……………

THANK YOU FOR YOUR TIME

Table S1.

Table 1. Univariate analysis of knowledge and attitudes towards SIV and COVID-19 vaccines/COVID-19 vaccination and SIV. (N=2592)

**Variables**	**Yes (%) Or Correct (%)**	**Vaccinated Yes/Correct (%)**	**Vaccinated No/Incorrect (%)**	**Proportional Ratio (PR) 95% CI**	**Sig.**
12. Do you belong to a vulnerable/high risk group due to your medical history?	426 (16.4)	334 (78.4)	1465 (67.7)	1.16 (1.09-1.23)	**<0.001**(C)
13. Do you live with older individuals or individuals belonging to a vulnerable/high risk group due to their medical history?	715 (27.6)	519 (72.6)	1280 (68.2)	1.06 (1.01-1.12)	**0.030**(C)
14a, 14b, 14c (Generic knowledge about vaccination)	981 (37.8)	799 (81.4)	1000 (62.1)	1.31 (1.25-1.38)	**<0.001**(C)
14a.The HPV vaccine is recommended for all males up to 18 years of age in the country	1900 (73.3)	496 (72.0)	1300 (68.4)	1.05 (0.995-1.11)	0.082(C)
14b. After the flu vaccination, certain foods are not permitted to be consumed for a period of 24 hours	1222 (47.1)	1072 (78.2)	727 (59.5)	1.32 (1.25-1.39)	**<0.001**(C)
14c. One of the contraindications of the flu vaccine is an allergy to eggs	1185 (45.7)	931 (78.6)	868 (61.7)	1.27 (1.21-1.34)	**<0.001**(C)
15a, 15b, 15c (Attitudes towards vaccination)	953 (36.8)	813 (85.3)	986 (60.2)	1.42 (1.35-1.49)	**<0.001**(C)
15a Vaccinations are an important tool for the protection of public health, and in particular for health professionals and workers in the health sector	2423 (93.5)	1743 (71.9)	56 (33.1)	2.17 (1.75-2.69)	**<0.001**(C)
15b. Natural immunity acquired via disease is always preferable to immunity acquired via vaccination	1231 (47.5)	1105 (81.4)	691 (56.1)	1.45 (1.37-1.53)	**<0.001**(C)
15c. Many vaccines often have serious side effects	1250 (48.2)	1088 (81.3)	708 (56.6)	1.44 (1.36-1.52)	**<0.001**(C)
19a,19b, 19c (Knowledge about COVID-19 vaccines)	1290 (49.8)	1045 (81.0)	754 (57.9)	1.40 (1.33-1.48)	**<0.001**(C)
19a. Some of the vaccines against SARS-CoV-2 which are approved and used in the country are based on mRNA technology	2311 (89.2)	1652 (71.5)	144 (52.0)	1.38 (1.22-1.54)	**<0.001**(C)
19b. The dosage regimen of the vaccines against SARS-CoV-2 includes 3 doses *	1807 (69.7)	1336 (73.9)	460 (59.0)	1.25 (1.18-1.34)	**<0.001**(C)
19c. There is evidence that mRNA technology interferes with the DNA of cells	1648 (63.6)	1293 (78.5)	503 (53.5)	1.47 (1.37-1.56)	**<0.001**(C)
20. Have you been or will you be vaccinated with any of the vaccines against the SARS-CoV-2 virus which causes COVID-19, which have received the necessary approvals from the European Medicines Agency and the National Medicines Agency?	2101 (81.1)	1621 (77.2)	177 (36.1)	2.14 (1.89-2.41)	**<0.001**(C)
*16. Are you the parent/guardian of one or more children?	1539 (59.4)	1083 (70.4)	716 (68.0)	1.04 (0.98-1.09)	0.198(C)
** Do you adhere to the child vaccination — YES, I vaccinate my children according to the National	1501 (97.9)	1065 (71.0)		2.20 (1.32-3.67)	**<0.001**(C)

program suggested by the National Vaccination Program in the country? (total 1533)	Vaccination Program					
	I select and carry out some vaccinations	31 (2.0)	10 (32.3)	Ref.		
	I do not vaccinate my children	1 (0.1)	0 (0.0)			
*From 2592 respondents, 1053 did not have a child, ** 1533 (99.6%) responded from 1539 individuals who had a child						

Figure S1 : Reasons for SIV and COVID-19 vaccine refusal

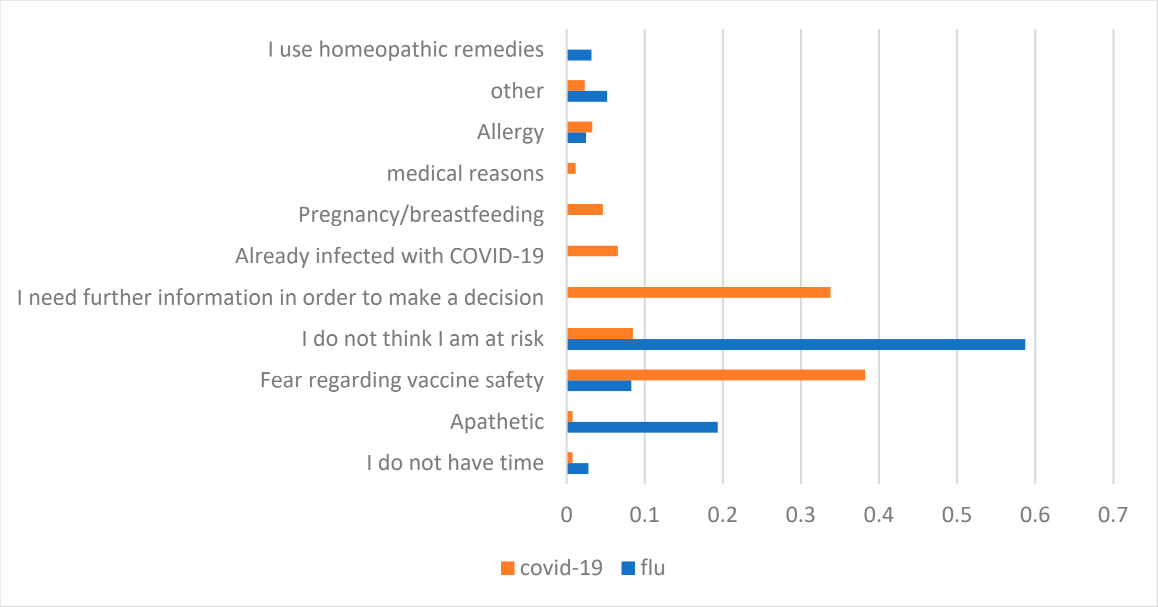

Supplement: Supplementary file 1 [file ijerph-20-06247-s001.zip › ijerph-2346019-supplementary.pdf]
